# Supplementary material for: Chromothripsis during telomere crisis is independent of NHEJ, and consistent with a replicative origin
Source: Genome Res. 2019 May;29(5):737–49. doi: 10.1101/gr.240705.118 (PMC6499312; doi:10.1101/gr.240705.118)
Supplement: Supplemental Material [file supp_gr.240705.118_Supplemental_file_1.zip › contigs/annotated_contigs/DB79/contig.2.DB79_length_266_mean_cov_14.5676691729.docx]

**DB79_length_266_mean_cov_14.5676691729**

```
GGGGGGCCCGGGGGGGGGGGGCGCGTCCGGCGCCGCCGTCCTTCCGGTTCGTCTCCCCCCCCCCGGGCCCC|CCCCGGGGGGGGGGGGG
                                                                        >chr2:223731463-
GGGGGGGGGGGGGGGGG|GGGGGGG|AGGGCGCGCGACCCCGGTCGGCGCGCCCCGCTTCTTCGGTTCCCGCCTCCTCCCCGTTCACCG
223731504 - E=2e-07 p=3e-02 >chrUn_gl000220:118545-118706 + E=9e-83
CCGGGGCGGCTCGTCCGCTCCGGGCCGGGACGGGGTCCGGGGAGCGTGGTTTGGGAGCCGCGGAGGCGGCCGCTCCGAGCCGGGCCCGT

GG
```
